# Lower Levels of Vitamin D Are Associated with Higher Vasoactive–Inotropic Scores in Major Cardiac Surgery

**DOI:** 10.3390/life14111349

**Published:** 2024-10-22

**Authors:** Adrian Stef, Constantin Bodolea, Simona Sorana Cainap, Monica Muntean, Aurelia Georgeta Solomonean, Nadina Tintiuc, Razvan Olimpiu Mada, Gabriel Cismaru

**Affiliations:** 1 Clinical Department of Anesthesia and Intensive Care, Heart Institute “Niculae Stancioiu”, “Iuliu Hatieganu” University of Medicine and Pharmacy, Motilor 19-21, 400001 Cluj-Napoca, Romania; stef.adrian@yahoo.com (A.S.); aureliageorgeta@gmail.com (A.G.S.); ntintiuc@yahoo.com (N.T.); 2 Anesthesia and Intensive Care 2 Discipline, “Iuliu Hatieganu” University of Medicine and Pharmacy, Victor Babes Nr 8 Street, 400012 Cluj-Napoca, Romania; cbodolea@gmail.com; 3 Department of Mother and Child, 2nd Pediatric Discipline, “Iuliu Hatieganu” University of Medicine and Pharmacy, Victor Babes Nr 8 Street, 400012 Cluj-Napoca, Romania; cainap.simona@gmail.com; 4 Infectious Diseases and Epidemiology Department, “Iuliu Hatieganu” University of Medicine and Pharmacy, Victor Babes Nr 8 Street, 400012 Cluj-Napoca, Romania; monica.muntean@umfcluj.ro; 5 Cardiology Department, Heart Institute “Niculae Stancioiu”, Motilor 19-21, 400001 Cluj-Napoca, Romania; mada_razvan@yahoo.com; 6 4th Department of Internal Medicine, Cardiology Rehabilitation, “Iuliu Hatieganu” University of Medicine and Pharmacy, Victor Babes Nr 8 Street, 400012 Cluj-Napoca, Romania

**Keywords:** vitamin D, cardiopulmonary bypass, major cardiac surgery, vasoactive–inotropic score, renal failure

## Abstract

Background: The vasoactive–inotropic score (VIS) predicts unfavorable outcomes after cardiac surgery in both children and adults. In our adult population, we investigated whether preoperative levels of vitamin D can predict the VIS and whether both vitamin D and the VIS can predict adverse outcomes following major heart surgery. Methods: Between 1 October 2021 and 28 February 2022, 300 patients underwent major cardiac surgery at our institution. Eighty-three of them had their 25-OH vitamin D levels measured before surgery. For this cohort, we calculated the VIS based on doses of vasoactive and inotropic medications administered post-surgery. Utilizing receiver operating curves, the predictive accuracy of vitamin D levels and the VIS in predicting acute kidney injury was assessed. Results: The median age of the cohort was 66 (IQR 61–71) years, with 59% being male and a median BMI of 28.4 (IQR 25.2–31.6). The most common procedures were aortic valve replacement, mitral valve replacement, coronary artery bypass grafting, aortic valve and ascending aorta repair, and ASD correction. There was a significant difference in the postoperative VIS between patients with vitamin D deficiency, i.e., <20 ng/mL, and patients with vitamin D values > 20 ng/mL (3.5 vs. 1.3 *p* < 0.04). We also found a significant correlation between the VIS and the days of hospitalization (r = 0.335; *p* = 0.002), the days of stay in the intensive care unit (r = 0.547; *p* < 0.00001), and the mechanical ventilation time (r = 0.327; *p* = 0.025). Both vitamin D levels and the VIS predicted postoperative acute kidney injury (*p* < 0.05). Conclusions: Vitamin D deficiency is correlated with the VIS in adults undergoing major cardiac surgery. Both vitamin D levels and the VIS can predict unfavorable postoperative outcomes.

## 1. Introduction

Unfavorable outcomes after major surgery can be predicted using scoring systems [1]. Nevertheless, EuroScore II, STS risk score, and Parsonnet score were specifically designed to predict the prognosis following cardiac surgery by considering preoperative factors rather than postoperative data [2,3,4]. The vasoactive–inotropic score (VIS) is determined by quantifying the combined effects of inotropes and vasoconstrictors for maintaining hemodynamic balance. A greater score indicates a more significant hemodynamic impairment. Gaies et al. [5], Wernovski et al. [6], and Favia et al. [7] established the validity of the VIS in predicting adverse outcomes in pediatric populations requiring heart surgery.

Previously conducted studies suggest that vitamin D is involved in vascular and cardiac function [8] and reduces the risk of arrhythmia [9]. A recent study showed no significant difference in the vitamin D supplementation group in terms of ICU stay, hospital stay, or duration of mechanical ventilation [10]. Nevertheless, a recent systematic review revealed contradictory findings concerning the overall survival and duration of stay in the intensive care unit (ICU) associated with vitamin D levels [11].

In our study, we investigated whether vitamin D levels and the VIS could predict unfavorable outcomes after major cardiac surgery in adult patients.

## 2. Materials and Methods

The present study was structured as a retrospective, single-arm, observational investigation conducted at a single cardiac surgery center and approved by the “Iuliu Hatieganu” University of Medicine Ethics Committee (approval number 259 on 28 September 2023). All patients who underwent major heart surgery (aortic or mitral valve procedures, coronary artery bypass, aortic valve and ascending aorta repair, aortic aneurysm repair, or association between CABG and other valve procedures) from 1 October 2021 to 28 February 2022 were selected for inclusion in the study, which specifically focused on patients who underwent preoperative evaluations of 25-OH vitamin D levels: The research was conducted in accordance with the principles of the Declaration of Helsinki and Good Clinical Practice Guidelines. The requirement to obtain patient consent was waived because the data analysis was retrospective.

The exclusion criteria included age <18 years, emergency need for surgery, patients with hemodynamic instability referred for major cardiovascular surgery, patients who had catheter-based transfemoral aortic valve replacement, aortic arch intervention, or descending aortic surgery, patients that took vitamin D supplements prior to surgery, and patients with a hospital length of stay less than 24 h (transferred to other cardiac facilities). After heart surgery, all patients were monitored in the ICU. The administration of inotropic drugs such as dobutamine, dopamine, epinephrine, norepinephrine, milrinone, and vasopressin was started in the case of reduced cardiac output associated with hypotension. Pharmacological responses to vasoactive and inotropic drugs were monitored, and the administration of these drugs was adjusted to the minimum necessary dosage rates to sustain sufficient cardiac output and blood pressure. The early postoperative use of vasoactive drugs can prevent multi-organ ischemia and dysfunction, and infusion was rapidly terminated or the rate decreased once the patient achieved circulatory stability. Vitamin D levels were correlated with VIS, cardiopulmonary bypass perfusion time, cross-clamp time, mechanical ventilation time, length of stay in the ICU, total length of stay in the hospital, and duration of mechanical ventilation.

The patients were registered in a single database, consisting of demographic, clinical, and postoperative information extracted from our internal hospital database. The data were transferred to an Excel spreadsheet and then to an SPSS file. All measurements for 25 OH vitamin D were conducted from venous blood using the Biotek Microplate 50 TS washer (Agilent Technologies Inc., Santa Clara, CA, USA) and the 800 TS reader (Agilent Technologies Inc., Santa Clara, CA, USA). Vitamin D deficiency was defined as a 25(OH)D3 level of <20 ng/mL; vitamin D insufficiency was defined as a 25(OH)D3 level of 20–30 ng/mL; and normal vitamin D levels were defined as a 25(OH)D3 level of at least 30 ng/mL. Postoperative hemorrhage, renal dysfunction following surgical intervention, food restrictions, and fluid infusions are known to alter the postoperative status of vitamin D. Therefore, our analysis exclusively considered preoperative vitamin D levels to assess whether they could serve as a predictor of the patient’s postoperative outcome.

For calculating the VIS, the patient’s medical record was reviewed to determine the administration rate of vasoactive medications and inotropes. The cutoff for a high and low VIS was a score of 15 points [12].

### Statistical Methods

Categorical variables were expressed as counts and proportions, while continuous variables were reported using the median together with ± the interquartile range. When suitable, correlations were conducted using Pearson or Spearman methodologies. Where suitable, Student’s T test or the Mann–Whitney U test were used to compare continuous variables. A comparison of categorical variables was conducted using the chi-square test. Outcomes following surgery were predicted using receiver operator curves (ROCs), which were also used to ascertain the specificity, sensitivity, and cutoff values of vitamin D and VIS for predicting unfavorable outcomes. All analyses were conducted using SPSS version 23.0 (IBM SPSS Inc., Chicago, IL, USA). Statistical significance was defined as *p*-values less than 0.05.

## 3. Results

Between 1 October 2021 and 28 February 2022, 300 patients underwent major cardiac intervention at our institution: aortic or mitral valve procedures, coronary artery bypass, aortic valve and ascending aorta repair, aortic aneurysm repair, or association between CABG and other valve procedures. Eighty-three of them had a 25-OH vitamin D measurement taken before surgery and were included in this study. The median age was 66 years (range 38–81); the median value of 25-OH vitamin D was 16.0 ng/mL (IQR 15.0–19.0); and the plasma level was below 20 ng/mL in 65 patients (78.3%) and >20 ng/mL in 18 patients (21.7%). No significant difference in sex (*p* = 0.536), age (*p* = 0.512), BMI (*p* = 0.634), days of hospitalization (*p* = 0.808), days of hospitalization in the ICU (*p* = 0.421), duration of mechanical ventilation (0.563), CPB time (*p* = 0.968), or clamping time (*p* = 0.569) was found between the two vitamin D groups. However, after excluding one outlier patient that had 44 days of hospitalization, the statistical analysis yielded a *p*-value of 0.77 for the difference in hospitalization days between the two vitamin D groups and a *p*-value of 0.042 for the difference in ICU stay.

However, there was a significant difference in the postoperative VIS between patients with vitamin D deficit values < 20 ng/zl and patients with normal vitamin D values > 20 ng/mL (3.5 vs. 1.3; *p* < 0.04) (Table 1). The vasopressors and inotropes used were dopamine in 6 patients (mean dose: 2.7 ± 0.7 mcg/kg/min), dobutamine in 27 patients (mean dose: 3.0 ± 1.8 mcg/kg/min), epinephrine in 3 patients (mean dose: 0.04 ± 0.02 mcg/kg/min), and norepinephrine in 26 patients (mean dose: 0.05 ± 0.09 mcg/kg/min).

To obtain the vasoactive–inotropic score (VIS), the weighted sum of all prescribed inotropes and vasoconstrictors was computed using the following formula:

VIS = dopamine (µg/kg/min) + dobutamine (µg/kg/min) + 100 × epinephrine (µg/kg/min) + 100 × norepinephrine (µg/kg/min) + 10 × milrinone (µg/kg/min) + 10,000 × vasopressin (units/kg/min) + 50 × levosimendan (µg/kg/min) [13]. The cutoff for a high and low VIS was a score of 15 points; therefore, we divided our cohort into two groups based on VIS < 15 or ≥15. There was no significant difference in age (*p* = 0.395), sex (*p* = 0.511), BMI (*p* = 0.942), CPB time (*p* = 0.456), clamping time (*p* = 0.372), or mechanical ventilation time (*p* = 0.735) between the two VIS groups according to the 15-point cutoff. However, patients with a VIS >15 had a prolonged stay in the hospital (16 ± 1.4 vs. 10 ± 4.9 days) and in the ICU (11 ± 8.4 vs. 3.7 ± 4.9 days) compared to patients with a VIS < 15 (*p* < 0.05) (Table 2).

We also found a significant correlation between the VIS and days of hospitalization (r = 0.335; *p* = 0.002), days of stay in the ICU (r = 0.547; *p* < 0.00001), and mechanical ventilation time (r = 0.327; *p* = 0.025).

The univariate regression analysis indicated that there was an association between age [OR 1.1; 95% CI 1.02–1.2; *p* = 0.013], VIS [OR 1.4; 95% CI 1.005–1.937; *p* = 0.04], CPB time [OR 1.015; 95% CI 1.003–1.03; *p* = 0.018], clamping time [OR 1.022; 95% CI 1.005–1.04; *p* = 0.01], and a longer ICU stay.

Variables found to be significantly associated with ICU stay in the univariate analysis (*p* < 0.05) were included in the multivariate regression analysis, which showed an association only between VIS and ICU stay. Patients with a high VIS had significantly greater odds of having a longer ICU stay [OR 1.4; 95% confidence interval (CI) 1.1–1.7; *p* = 0.005].

We considered two groups based on major surgery: low complexity (single aortic or mitral valve procedures, coronary artery bypass) and high complexity (aortic valve + ascending aorta repair, aortic aneurysm repair, association between CABG + valve procedures, multiple-valve procedures).

We compared the two groups, low complexity vs. high complexity, and found significant differences in terms of VIS (2.9 vs. 6.5; *p* = 0.01), duration of mechanical ventilation (5.7 vs. 8.2 h; *p* = 0.04), ICU stay (3.4 vs. 5.9 days; *p* = 0.04), days of hospitalization (10.2 vs. 12.5; *p* = 0.05), CPB time (83 vs. 147 min; *p* = 0.001), and clamping time (63 vs. 120 min; *p* = 0.0001).

We compared patients with vitamin D deficit values < 20 ng/zl and patients with normal vitamin D values > 20. In the high-complexity group, we found a significant difference in postoperative VIS (6.2 vs. 8.0; *p* = 0.04), mechanical ventilation time (9.2 vs. 3.6 h; *p* = 0.004), ICU stay (6.5 vs. 3.0; *p* = 0.421), days of hospitalization (12.8 vs. 11 *p* = 0.03), CPB time (156 vs. 105 min; *p* = 0.01), and clamping time (128 vs. 79; *p* = 0.01). However, there was no significant difference in the low-complexity group (*p* > 0.60 for all tests).

**Figure 1:**
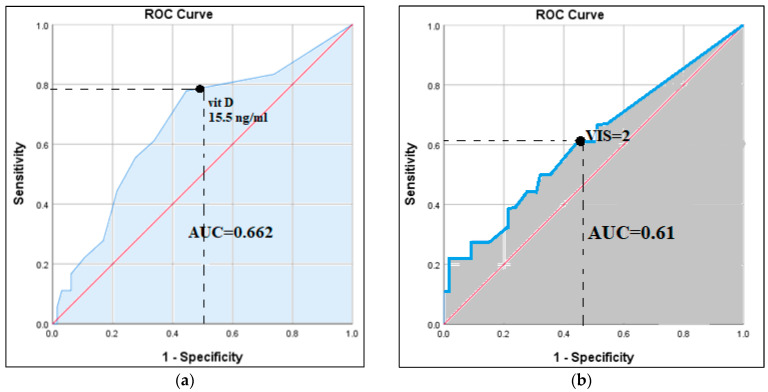
Receiver operator curves for vitamin D and VIS as predictors of postoperative acute kidney injury. (**a**) Vitamin D levels predict postoperative acute kidney injury—AUC: 0.662. A value of 15.5 ng/mL predicts development of renal failure with a sensitivity of 78% and specificity of 56%. (**b**) VIS predicts postoperative acute kidney injury—AUC: 0.61. A value of 2 ng/mL predicts development of acute kidney injury with a sensitivity of 61% and specificity of 56%.

Using receiver operating curves (ROCs), we found that vitamin D levels predicted postoperative acute kidney injury (AUC, 0.662; 95% CI, 0.52–0.80; *p* = 0.03; Figure 1a). A level below 15.5 ng/mL predicted the occurrence of acute kidney injury with a sensitivity of 78% and specificity of 56%. The VIS also predicted postoperative acute kidney injury (AUC, 0.61; 95% CI, 0.45–0.77; *p* = 0.048; Figure 1b). A value of VIS = 2 predicted the occurrence of acute kidney injury with a sensitivity of 61% and specificity of 56%.

## 4. Discussion

The main finding of this retrospective cohort study was that both vitamin D levels and the VIS were significant predictors of unfavorable outcomes following major heart surgery in a sample of selected adults. Patients with a VIS > 15 had a prolonged stay in the hospital (16 vs. 10 days) and in the ICU (11 vs. 3.7 days) compared to those with a VIS < 15. Previous research supports the prognostic value of a VIS > 15. Gaies et al. [11] calculated the optimal cutoff value for the vasoactive–inotropic score to predict morbidity and mortality in infants after cardiopulmonary bypass. According to the authors, moderate cardiovascular support on admission to the ICU after congenital heart surgery typically includes vasoactive infusions at dosages that result in a VIS of approximately 15. Based on this value, they considered a high VIS to be above 15 and a low VIS to be below 15. In a study by Sanil et al. [12] on children undergoing a heart transplant, 51 patients were divided into two groups based on the VIS: high VIS ≥ 15 and low VIS < 15. A high VIS > 15 was independently associated with increased short-term morbidity after pediatric heart transplant. In a study by Perez-Navaro et al. [13], a VIS > 15.5 had a high specificity (92.87%; 95% CI: 86.75–98.96) and increased negative predictive value (75.59%, 95% CI: 71.1–88.08) for low cardiac output syndrome after congenital heart disease surgery. This value was an independent predictor of unfavorable outcomes, and the predictive value was not increased if cardiac biomarkers were added to the VIS.

Adults experiencing vitamin D deficiency typically require more vasoactive/inotropic support during extended hospitalizations. Prior research has demonstrated that the VIS provides good predictive accuracy and discriminatory capacity for infant and pediatric patients following cardiac surgery [11,12,13,14,15]. Sanil and Aggarwal [12] assessed the VIS following 51 consecutive open-heart transplant procedures. Patients with a VIS > 15 exhibited significantly prolonged ICU stays, increased inotropic requirements, extended ventilatory durations, and a higher incidence of short-term morbidities. Gaies et al. [11] conducted a study including 391 newborns and concluded that a high VIS greater than 15 was substantially correlated with 30-day mortality, longer duration of mechanical respiration, and longer length of ICU stay. Davidson et al. [16] showed that an elevated VIS after cardiothoracic surgery was significantly correlated with extended mechanical ventilation duration, prolonged stays in intensive care units, and increased total hospital stay in neonates and infants. Our research on adults reveals similar results to those of the previously described studies conducted on children. Koponen et al. [17] also showed that the VIS demonstrates comparable predictive accuracy and discriminatory abilities in an adult population as in children. The causal association between elevated doses of vasoactive–inotropes and adverse outcomes is difficult to ascertain clearly. Elevated dosages of vasoactive drugs may only reflect cardiovascular morbidity rather than direct worsening of the clinical state of the patient.

Our investigation found no statistically significant association between VIS and mortality. The findings of our study align with a recent meta-analysis that found no association between inotropes and increased mortality in the general population [18].

Our research identified the VIS as a promising tool for the prediction of acute kidney injury. The VIS showed good predictive value for acute kidney injury, with an AUC of 0.61, a sensitivity of 61%, and a specificity of 56% for a cutoff value of 2. One possible strategy would be to decrease the dosage of inotropic and vasoactive drugs to a level that enables adequate tissue perfusion without risking adverse effects on renal perfusion. On the contrary, it is crucial to promptly address factors that may elevate the VIS, such as an infection that may result in infectious shock requiring vasoactive medications or an untreated coronary lesion that may lead to myocardial necrosis, reducing the ejection fraction to cardiogenic shock requiring inotropes [19].

Vitamin D deficiency is defined as a 25(OH) vit D level below 20 ng/mL and vitamin D insufficiency as a level of 21–29 ng/mL. In our cohort, patients with vitamin D deficiency had a higher VIS compared to patients with levels > 20 ng/mL (3.5 vs. 1.3). Our findings are consistent with those of Ye et al. [20], who demonstrated that vitamin D deficiency was linked to an increased need for inotropic support in children who underwent cardiac surgery for congenital heart disease. Their study revealed that 33% of children with vitamin D levels < 20 ng/mL had a VIS above 15, and 22.8% of children with vitamin D levels of >20 ng/mL had a VIS greater than 15 (*p* = 0.000). To the best of our knowledge, our study is the first to demonstrate an association between vitamin D deficiency and VIS in adult patients requiring major heart surgery.

Ye et al. [11] also showed that children with vitamin D levels < 20 ng/mL have longer CBP times (67 min) and clamping times (37 min) compared to children with vitamin D levels > 20 ng/mL (52 min for CBP time and 29 min for clamping time). However, in our cohort of adults, we found no difference in CBP time and clamping time between patients with levels < 20 ng/mL and patients with levels > 20 ng/mL.

We also found a significant correlation between VIS and duration of mechanical ventilation. Despite significant advancements in perioperative care for cardiac surgery, the prevalence of extended mechanical ventilation in postoperative patients remains as high as 22% [21]. Low levels of vitamin D, <20 ng/mL, were demonstrated to be linked to prolonged mechanical ventilation and extended hospital and intensive care unit stays in children hospitalized in an ICU [22,23,24]. De Pascale et al. [25] demonstrated that critically ill septic patients with extremely low vitamin D levels, <7 ng/mL, at ICU admission may experience adverse outcomes, including increased mortality and elevated infection rates. Deficiency in vitamin D has been associated with worse outcomes in patients hospitalized in the ICU and negative outcomes for infectious diseases [26].

Vitamin D is crucial for the control of the immune system as it decreases the inflammatory response, increasing phagocytosis, and stimulates lymphocyte proliferation. Vitamin D deficiency inhibits B cell proliferation and differentiation, prevents immunoglobulin release by B cells, and inhibits T cell proliferation [27]. Vitamin D deficiency leads to diminished production of inflammatory cytokines, including IL-1, IL-6, IL-12, IL-21, and TNF-α, while enhancing the production of anti-inflammatory cytokines, such as IL-10 [28,29]. Vitamin D acts by improving the absorption of calcium and phosphorus in the intestinal tract, promoting the mobilization of calcium from bones, and augmenting the renal reabsorption of calcium in the distal tubules [30]. Vitamin D exerts its influence on cardiac contractility via the vitamin D receptor (VDR) located on myocardial cells, interacting at the molecular level to modify myocardial contractility in both healthy and diseased hearts [31]. Cellular vitamin D receptors are present in all cardiac cells, and low vitamin D levels may directly affect atrial and ventricular cardiac myocytes. Therefore, vitamin D supplementation might improve outcomes after major cardiac surgery and decrease the use of inotropic and vasoactive agents. Whether the supplementation of vitamin D after cardiac surgery can improve outcomes and lower hospital and ICU stays remains controversial [32], and extensive prospective trials on the effectiveness of vitamin D supplementation are required.

Our findings suggest, as therapeutic implications, that preoperative vitamin D administration may elevate serum 25OH vitamin D levels and prevent unfavorable outcomes, such as prolonged mechanical ventilation, extended ICU stay, prolonged hospitalization, and acute kidney injury. This can be accomplished through the oral administration of a high dose of vitamin D, as demonstrated in a study by Leszczyńska et al. [33], which utilized a single dose of 120,000 IU of vitamin D3, leading to an increase in levels to 20–50 ng/mL. An intravenous bolus dose of 50,000–100,000 U, as demonstrated in research by Barker et al. [34], Armas et al. [35], Ilahi et al. [36], or Heaney et al. [37], may be more efficient in quickly increasing vitamin D levels in patients who need an immediate elevation in serum vitamin D levels before major cardiac surgery.

### Limitations

This study is limited by its observational nature, which, despite multivariable adjustments for multiple potential confounders, predisposes it to residual and unobserved confounding. Its observational nature also precluded the establishment of causality. Additionally, the relatively low event rates for some of the outcomes reduced the reliability of our findings, which cannot be applied broadly because of the limited sample size (n = 83). We believe that an increased patient population in a multicenter study would have yielded a more rigorous statistical result. Another important limitation of this study was its single-center design, which may introduce biases related to certain surgical techniques or patient demographics. For different cardiac surgery units that may have variations in terms of vasoactive and inotropic drug treatment regimens and the availability of mechanical cardiovascular support devices, adjustment of the VIS cutoff may be necessary. Further, larger prospective studies addressing the above issues are warranted to offer more proof of the link between vitamin D levels and surgical outcomes.

## 5. Conclusions

The vasoactive–inotropic score was shown to be associated with preoperative vitamin D levels. Patients with vitamin D levels below 20 ng/mL had greater postoperative VISs compared to patients with levels over 20 ng/mL. A VIS above 15 was linked to an extended duration of hospitalization and a prolonged stay in the ICU. A significant correlation was seen between the VIS and the duration of hospitalization, the days of stay in the intensive care unit, and the duration of mechanical ventilation. Both vitamin D levels and the VIS were predictive of postoperative acute kidney injury.

## Figures and Tables

**Table 1:** Comparison between 2 vitamin D groups: low levels < 20 ng/mL and normal levels > 20 ng/mL.

Parameter	Total	25OHvitD ≤ 20 ng/mL	25OHvitD > 20 ng/mL	*p*-Value
Age, years, median (IQR)	66 (61–71)	66.5 (61.7–69.2)	65 (60.5–71.5)	0.536
BMI, median (IQR)	28.4 (25.2–31.6)	28.7 (25.6–30.4)	29.0 (25.0–31.7)	0.634
CPB time (min), median (IQR)	101 (68.5–124)	102 (73–125)	100 (67–125)	0.968
Clamping time (min), median (IQR)	74 (45–93)	69 (49.5–83.2)	77 (39.5–96)	0.569
VIS median (IQR)	1.3 (0–3.5)	0 (0–2.5)	2.0 (0–4.0)	**0.04**
Mechanical ventilation time	5.0 (3.0–8.0)	5.0 (3.0–8.2)	5.0 (3.0–8.0)	0.563
Hospitalization duration (days)	9.0 (8.0–12.0)	10.0 (8.0–12.0)	9.0 (8.0–12.0)	0.421
Intensive care unit stay (days)	3.0 (2.0–4.0)	3.0 (2.0–4.0)	3.0 (2.0–4.0)	0.808

IQR = interquartile range; CPB = cardiopulmonary bypass time; VIS = vasoactive–inotropic score. Red color and bold means significant *p* value < 0.05

**Table 2:** Comparison between 2 VIS groups: low score < 15 and high score > 15.

Parameter	Total	VIS < 15	VIS ≥ 15	*p*-Value
Age, years, median (IQR)	66 (61–71)	66.5 (61.7–69.2)	65 (60.5–71.5)	0.395
BMI, median (IQR)	28.4 (25.2–31.6)	28.7 (25.6–30.4)	29.0 (25.0–31.7)	0.942
CPB time (min), median (IQR)	101 (68.5–124)	102 (73–125)	100 (67–125)	0.456
Clamping time (min), median (IQR)	74 (45–93)	69 (49.5–83.2)	77 (39.5–96)	0.372
Mechanical ventilation time	5.0 (3.0–8.0)	5.0 (3.0–8.2)	5.0 (3.0–8.0)	0.735
Hospitalization duration (days)	9.0 (8.0–12.0)	9.0 (8.0–12.0)	10.0 (8.0–12.0)	0.049
Intensive care unit stay (days)	3.0 (2.0–4.0)	1.5 (1.0–2.0)	3.0 (2.0–4.0)	0.04

IQR = interquartile range; CPB = cardiopulmonary bypass time; VIS = vasoactive–inotropic score. Red color means significant *p* value < 0.05

## Data Availability

Data supporting the reported results can be found in Mega cloud through the following link: https://mega.nz/fm/yYwzSS6a (accessed on 19 September 2024).
